# How Does a Porcine Herpesvirus, PCMV/PRV, Induce a Xenozoonosis

**DOI:** 10.3390/ijms26083542

**Published:** 2025-04-09

**Authors:** Joachim Denner

**Affiliations:** Institute of Virology, Free University, 14163 Berlin, Germany; joachim.denner@fu-berlin.de

**Keywords:** porcine cytomegalovirus/porcine roseolovirus (PCMV/PRV), xenozoonosis, consumptive coagulopathy, human herpesvirus 6 (HHV-6), coagulation

## Abstract

Porcine cytomegalovirus/porcine roseolovirus (PCMV/PRV), a porcine herpesvirus, has been shown to significantly reduce the survival time of porcine xenotransplants in non-human primates. The virus was detected in all the examined organs of baboons transplanted with PCMV/PRV-positive organs and it was also transmitted to the first human recipient of a pig heart, contributing to the patient’s death. PCMV/PRV induces consumptive coagulopathy and thrombocytopenia in xenotransplant recipients. Initial studies in baboons revealed that the virus triggered increased release of tumor necrosis factor α (TNFα) and interleukin 6 (IL-6), along with elevated levels of tissue plasminogen activator (tPA) and plasminogen activator inhibitor 1 (PAI-1) complexes. Since there is no evidence that PCMV/PRV infects primate cells, including human cells, the virus appears to directly interact with immune and endothelial cells, disrupting cytokine signaling and coagulation pathways. The highest viral load was detected in the explanted pig heart, suggesting active replication at this site. Additionally, cells expressing PCMV/PRV proteins were identified in all the examined baboon organs, where pig cells were also found. Since PCMV/PRV affects only xenotransplant recipients and not healthy humans, this condition should be classified as a xenozoonosis. Interestingly, antibodies against human herpesvirus 6 (HHV-6) cross-react with PCMV/PRV and may contribute to protection against infection in humans. Further research is needed to uncover the molecular mechanisms underlying this xenozoonotic disease.

## 1. PCMV/PRV—Molecular Biology

Porcine cytomegalovirus/porcine roseolovirus (PCMV/PRV) is a porcine herpesvirus in the genus *Roseolovirus,* not in the genus *Cytomegalovirus* [1,2]. The name given by the International Committee on Taxonomy of Viruses (ITCV) is suid betaherpesvirus 2 (SuBHV2), indicating that it belongs to the subfamily Betaherpesvirinae [3]. The misleading originally chosen name PCMV can be explained by reports on the appearance of cytomegalic cells with characteristic basophilic intranuclear inclusion bodies in the mucosal glands of the turbinates of pigs [4]. Sequence comparisons clearly identified PCMV/PRV as a roseolovirus closely related to the human roseoloviruses, human herpesvirus 6 (HHV-6) and HHV-7. The abbreviation PCMV/PRV should emphasize this point. HHV-6 and HHV-7 are widespread in the human population, and PCMV/PRV is also highly prevalent in pigs. For instance, a study on German slaughterhouse pigs found that all the animals were infected [5].

The virus has a linear double-stranded DNA genome 128,367 bp long and containing 79 open reading frames (ORFs) [2]. Of these ORFs, 69 have counterparts in HHV-6A, HHV-6B, and HHV-7. The genome is a direct repeat (DR)-unique (U)-DR type, similar to HHV-6A, HHV-6B, and HHV-7, but the PCMV/PRV DR is shorter and lacks predicted genes and telomeric repeats (TMRs). The absence of these TMR sequences means that PCMV/PRV, unlike the closely related HHV-6, cannot integrate into the host cell genome [2]. As a result, PCMV/PRV, like most other herpesviruses, has to maintain its genome as a circular episome during the latent stage of infection.

It is still unclear whether PCMV/PRV can infect cells from non-human primates and humans. Herpesviruses were once believed to be strictly species-specific, incapable of infecting other species. However, this understanding has evolved, with evidence now confirming transspecies transmission of several herpesviruses (for review see Ref. [6]). One example is baboon cytomegalovirus (BaCMV), which was shown to infect human cells in vitro and which was found in human recipients of baboon liver [7,8]. Furthermore, human cytomegalovirus (HCMV) was shown to infect pig cells [9]. Regarding PCMV/PRV and human cells, one study has reported infection in human cells [10], whereas one other has found no evidence of infection [11]. Further studies are needed to determine whether PCMV/PRV can infect primate cells, particularly undifferentiated cells such as stem cells.

## 2. Pathogenesis in the Pig

The primary route of infection is transmission from an infected mother sow, occurring after the maternal antiviral antibody levels decline in the piglet [12]. Unlike in many other mammals, these antibodies are not transferred via the placenta in pigs but are instead provided through colostrum. If the mother is infected, the piglet typically experiences only mild clinical symptoms and recovers quickly. However, if the piglet is born to a PCMV-negative mother and lacks partial protection by maternal antibodies, a more severe respiratory disease may develop, potentially leading to fatal outcomes. PCMV/PRV is occasionally associated with inclusion body rhinitis and pneumonia in piglets, reproductive disorders in pregnant sows and respiratory disease complex in older pigs [12]. The virus is shed in nasal secretions and it has also been detected in ocular secretion, urine, cervical fluid, and semen [13]. PCMV/PRV-infected sows are prone to abortion, with pathological changes including edema in the heart, lungs, and lymph nodes [14]. In production facilities, infections are generally asymptomatic due to the development of herd immunity. However, Chinese scientists mention that PCMV/PRV has caused huge economic losses to the porcine breeding industry [13].

PCMV/PRV is an immunosuppressive virus that mainly inhibits the immune function of the macrophage and T cell lymphatic systems [15]. Infections in pigs are often associated with opportunistic bacterial infections based on the immunosuppression by the virus. Transcriptome analysis of PCMV/PRV-infected pig thymuses showed that 2161 genes were upregulated and 3421 were downregulated compared with the uninfected group [15]. Among others, interleukin 1α (IL-1α), IL-1β, and IFN-α, were elevated in expression, whereas IL-12B, tumor necrosis factor (TNF), and transforming growth factor β1 (TGF-β1) were found to be consistently downregulated, as shown by qPCR, Western blot, and microarray analysis. IL-7, Il-8, and IL-15 were found to be upregulated, as shown by PCR and microarray. The expression levels of most genes involved in the T cell receptor (TCR) signaling pathway were downregulated. TGF-β is an immunosuppressive cytokine, and the activated TGF-β signaling pathway may function in PCMV/PRV infection as it does during infections by other immunosuppressive viruses, e.g., HCMV [16]. When microRNA (miRNA) expression profiles of PCMV/PRV-infected porcine macrophages via high-throughput sequencing were analyzed, 239 miRNA database-annotated and 355 novel pig-encoded miRNAs were detected. Of these, 130 miRNAs showed significant differential expression between the PCMV/PRV-infected and uninfected porcine macrophages [17]. When porcine small-RNA transcriptomes of PCMV/PRV-infected and uninfected organs were characterized by high-throughput sequencing, 92, 107, 95, 77, and 111 miRNAs were significantly differentially expressed in the lung, liver, spleen, kidney, and thymus after PCMV infection, respectively [18].

PCMV/PRV is able to infect and propagate in pig monocyte-derived macrophages (MDMs) in vitro. Infection decreased the expression of IL-8 and TNF-α and increased the expression of IL-10 on the mRNA transcription level [19].

Additionally, when primary porcine aortic endothelial cells (PAECs) were infected with PCMV/PRV in vitro, an increase in porcine tissue factor (TF) was observed, indicating virus-induced endothelial cell activation [20]. TF is also called coagulation factor III; it is present in subendothelial tissue and leukocytes and plays a major role in the coagulation initiating of thrombin formation from the prothrombin.

## 3. Pathogenesis in Non-Human Primate Xenotransplant Recipients

PCMV/PRV was transmitted in numerous preclinical xenotransplantation trials involving non-human primates. In the first reported case, pig thymokidneys were transplanted into baboons, leading not only to the transmission and replication of PCMV/PRV but also to the activation of BaCMV [21].

In eight preclinical trials involving the transplantation of pig thymokidneys, hearts, kidneys, and livers into baboons and cynomolgus monkeys, the transmission of PCMV/PRV to baboons or cynomolgus monkeys led to consumptive coagulopathy and thrombocytopenia, which were linked to a significantly reduction in survival time (for review, see Ref. [6]). Consumptive coagulopathy (or disseminated intravascular coagulation) is a pathological condition in which widespread activation of the coagulation system leads to excessive clot formation (thrombosis) and the simultaneous depletion of clotting factors and platelets, resulting in an increased risk of bleeding. Ongoing consumption of coagulation proteins and platelets leads to both clotting and hemorrhagic complications. Blood clots are formed throughout the body and block mainly small blood vessels [22]. Thrombotic microangiopathy (TMA) is a syndrome defined by the presence of endothelial damage, leading to the abnormal activation of coagulation, thrombocytopenia, vascular dysfunction, and organ damage [23].

A significant reduction in the survival time of orthotopically transplanted hearts from genetically modified pigs in baboons was also observed when PCMV/PRV-positive hearts were transplanted. Animals with a PCMV/PRV-positive heart did not survive beyond 30 days, whereas those with virus-negative pig hearts achieved survival times of up to 195 days [24,25]. In one case, PCMV/PRV was transmitted to the recipient baboon despite the virus not being detected in the donor pig [26]. In this case, PCMV/PRV was not detected in the donor pig’s blood by PCR due to viral latency, yet it triggered virus-specific clinical symptoms in the recipient.

In baboons transplanted with PCMV/PRV-positive hearts, the highest viral load was detected in the explanted pig heart, which was higher compared with the virus load in the donor pig organs [25]. This indicates that the primary replication of PCMV/PRV occurs in the transplanted pig organ, likely facilitated by the absence of the pig’s immune system, which normally controls viral replication in the donor. Additionally, the intensive pharmaceutical immunosuppression required for xenotransplantation further contributes to uncontrolled viral replication.

PCMV/PRV was found in all the examined baboon organs. This was shown by PCR and immunohistochemistry using specific antiviral antibodies [27]. In parallel, in all the examined baboon organs, indications for the presence of pig cells (microchimerism) were found using PCR, detecting porcine cellular gene sequences such as porcine short interspersed nuclear sequences (SINEs), which have hundreds of thousands copies in the pig genome [28]. Therefore, the virus-protein-positive cells were likely disseminated pig cells.

Analyzing the cytokine levels in the blood or baboon recipients, an increase in IL-6 and TNFα in the baboons with PCMV/PRV-positive hearts was observed [25]. IL-6 is a multifunctional cytokine that plays both pro-inflammatory and anti-inflammatory roles; it regulates inflammation and the development, maturation, and activation of T cells, B cells, and plasma cells [29]. Increasing IL-6 forms part of alloantibody generation through the stimulation of B cells in allotransplantation [30]. Blocking the interaction of IL-6 with its receptor leads to significant reduction in antibody-mediated rejection, in antibody production by splenic and bone marrow plasma cells, and by the induction of T regulatory cells. TNF induces inflammation and triggers cell death [31]. It acts on the immune system by the activation of white blood cells, blood coagulation, and the secretion of cytokines, as well as on endothelial cells, stimulating inflammation and coagulation. For example, TNF is able to stimulate the endothelial cells that form capillaries to activate blood clot formation within the capillaries to help prevent microbes from entering the bloodstream [32]. No alterations were observed in the serum levels of interferon γ (IFNγ), IL-2, IL-4, IL-5, and IL-10 [25].

## 4. Transmission to a Human Pig Heart Recipient

PCMV/PRV was transmitted to the first patient who received a genetically modified pig heart and contributed to the death of the patient [33,34]. The cardiac xenotransplant from a ten-gene modified pig sustained life despite the recipient’s pre-existing conditions and multiple surgical and non-surgical complications until the patient died from transplant failure on postoperative day 60. Clinical symptoms similar to the ones observed in the baboon with the PCMV/PRV-positive pig hearts were observed: at postoperative day 50, the endomyocardial biopsy revealed damaged capillaries with interstitial oedema, red cell extravasation, rare thrombotic microangiopathy, and complement deposition [33]. Endothelial changes ranged from prominent nuclei to cell swelling with areas of complete vascular dissolution [33]. The patient’s viral load continued to rise despite antiviral treatment. However, these antiviral drugs are very effective against HCMV but not against the roseolovirus PCMV/PRV [35]. Additionally, the reactivation of latent PCMV/PRV in the xenotransplant may have triggered a harmful inflammatory response in the patient, comparable with increased release of IL-6 in the baboons [25].

The donor pig for this patient was tested using a nasal swab, followed by PCR analysis to detect PCMV/PRV. However, this approach is flawed for two key reasons. First, rhinitis caused by PCMV/PRV is typically observed in young piglets infected by their mothers, but not in older pigs, such as the one used for this transplantation, making nasal swabs an ineffective diagnostic tool. Second, like many herpesviruses, PCMV/PRV can enter a latent state, rendering it undetectable in the blood and various organs where it may be hiding. To ensure accurate screening, immunological methods should have been employed, as previously described [36,37].

## 5. Molecular Insights into PCMV/PRV-Induced Xenozoonosis

**Figure 1:**
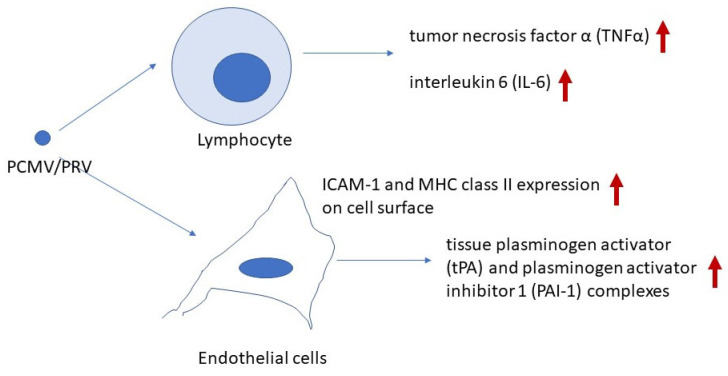
Effect of PCMV/PRV on endothelial and immune cells in recipients of PCMV/PRV-positive pig organs. The arrows indicate upregulation of expression.

Currently, two primary effects of PCMV/PRV on xenotransplant recipients can be proposed: first, an impact on the coagulation system, and second, an effect on the immune system (Figure 1).

The mechanism by which PCMV/PRV induces endothelial cell activation, leading to the early and severe loss of xenotransplants, remains partially unclear. In one study, transmission of PCMV/PRV was associated with increased expression of intercellular adhesion molecule-1 (ICAM-1) and major histocompatibility complex (MHC) class II in the transplant, indicating endothelial cell activation [38].

For comparison, an in vitro study demonstrated that human umbilical vein endothelial cells (HUVECs) infected with HCMV exhibited a significant increase in ICAM-1 surface expression [39]. An association between HCMV infections and the occurrence of rejection after renal allotransplantation is well known. HCMV-infected human proximal tubular epithelial cells (PTECs) also displayed increased levels of ICAM-1 and this was a direct effect requiring infectious virus [40].

Although PCMV/PRV is classified as a roseolovirus rather than a cytomegalovirus like HCMV, a similar upregulation of ICAM-1 and MHC class II antigens was observed on the endothelial surface of PCMV/PRV-positive pig kidney xenotransplants, but not in PCMV/PRV-negative kidneys [38]. The increased ICAM-1 expression on endothelial cells in infected xenotransplants facilitated the adhesion of activated lymphocytes and platelets. This profound endothelial activation, particularly in tubular capillaries, may contribute to interstitial hemorrhage and subsequent transplant failure [38]. Additionally, when primary porcine aortic endothelial cells (PAECs) were infected with PCMV/PRV in vitro, an increase in porcine tissue factor (TF) was observed, indicating virus-induced endothelial cell activation [20].

While it remains possible that PCMV/PRV can infect and damage non-human primate and human cells (see above), a viral protein or proteins could also interact with target cells in transplant recipients to induce or contribute to pathogenesis after xenotransplantation.

It is not uncommon for viral proteins to interact independently of viral infection on target cells. This applies to both regulatory and structural proteins. The human immunodeficiency virus 1 (HIV-1) protein Tat (trans-activating factor) is an example of a regulatory protein that acts not only endogenously within infected cells but also exogenously on uninfected ones. Extracellular Tat interacts with various cellular membrane receptors and can penetrate host cells through endocytic pathways [41]. Such an interaction has been shown to induce neuronal death [42]. Since PCMV/PRV encodes a CC chemokine (U83) [2], it may also produce non-structural proteins capable of interacting with the recipient’s target cells. Retroviral transmembrane envelope proteins illustrate how structural proteins can interact with uninfected cells and trigger immunosuppression (for review, see Ref. [43]). By binding to unidentified receptors on immune cells, retroviruses, their transmembrane envelope proteins, and synthetic peptides corresponding to a highly conserved domain among all retroviruses, the so-called immunosuppressive domain, stimulate the release of several cytokines, including IL-1β, IL-10, IL-6, IL-8, monocyte chemoattractant protein (MCP)-1 and MCP-2, tumor necrosis factor—α (TNF-α), and macrophage inflammatory proteins (MIP)-1α and MIP-3. Conversely, they suppress the expression of IL-2 and chemokine (C-X-C motif) ligand 9 (CXCL-9, also known as monokine induced by gamma interferon, MIG) [44].

Protein–protein interaction studies or in vitro assays demonstrating the interaction between PCMV/PRV proteins and human immune or endothelial cells are necessary to better understand the mechanism of how PCMV/PRV acts on endothelial and immune cells and contributes to the transplant rejection.

The genes and proteins of PCMV/PRV are not well studied. In the unique region, 79 genes were predicted and the majority of these genes have homologs in HHV-6A, HHV-6B, and HHV-7 in amino acid composition and sequence [2]. Furthermore, two pp65 proteins of PCMV have been identified that might have a similar function to the pp71 (UL82) and pp65 (UL83) of HCMV, which can interact with different cellular proteins that regulate gene expression and host immune response.

Finally, it is worth emphasizing the following point: zoonoses are defined as infectious diseases caused by a microorganism from a non-human vertebrate. PCMV/PRV is not considered a zoonotic virus when in contact with healthy individuals; however, it can cause disease in the context of xenotransplantation, when introduced into the recipient’s body through the xenotransplant; therefore, it should be classified as a xenozoonotic virus [45].

## 6. PCMV/PRV and HHV-6

HHV-6 and HHV-7 are the closest relatives of PCMV. When we screened butchers and blood donors for antibodies against PCMV/PRV, using a Western blot assay and a recombinant part of the gB protein of PCMV/PRV as antigen, antibodies against this protein were found in several individuals [46]. A detailed analysis showed that these antibodies are antibodies directed against HHV6, i.e., sera from humans infected with HHV-6 and carrying antibodies reacting in an immunofluorescence assay using HHV-6 strain U1102-infected cells recognized the recombinant PCMV/PRV protein used in our Western blot assay. Conversely, pig sera reacting against PCMV also reacted with human cells infected with HHV-6. We also analyzed a human IgG preparation (Cytotect) produced for the prophylaxis of HCMV infections for patients under immunosuppressive therapy, especially transplant recipients. These humane IgG preparations also reacted against the PCMV/PRV protein in our Western blot assay. Although derived from HCMV-positive donors, the reactivity with the PCMV protein can certainly be explained by the presence of antibodies against HHV-6 in the preparation. The prevalence of HHV-6 infection in the human population is very high.

Another situation suggesting a potential connection between anti-HHV-6 and anti-PCMV/PRV antibodies arises from the case of the first patient to receive a pig heart transplant. Interestingly, this patient was treated with cidofovir and intravenous immunoglobulin (IVIG) [33]. IVIG was administered due to severe hypogammaglobulinemia and its well-documented benefits in allotransplantation. However, it remains uncertain whether the temporary decrease in the otherwise steadily increasing PCMV/PRV load resulted from antiviral treatment or the presence of HHV-6 antibodies within the IVIG preparation [35]. IVIG likely contains antibodies against HHV-6, as it is derived from the plasma of 1000 to 100,000 donors [47], and HHV-6 is highly prevalent in the human population. In total, 90% of adults are latently infected with HHV-6 [48]. On day 49, the patient was treated again with cidofovir, but this time without IVIG, and no reduction in virus load was observed [33].

## 7. Conclusions

PCMV/PRV is a xenozoonotic virus that significantly shortens the survival of pig xenotransplants in non-human primates. It was also transmitted to the first human recipient of a pig heart, contributing to the patient’s death. Although it is still unknown whether PCMV/PRV infects primate cells, there are several indications that the virus interacts with immune and endothelial cells without infection, disrupting cytokine signaling and coagulation pathways. Further research is needed to uncover the molecular mechanisms underlying this xenozoonotic disease. Identifying the viral protein(s) responsible for the interactions between virus and xenotransplant recipient would be highly valuable.

## Data Availability

No new data were created.

## Abbreviations

The following abbreviations are used in this manuscript:

BaCMV	baboon cytomegalovirus
CXCL-9	chemokine (C-X-C motif) ligand 9
HCMV	human cytomegalovirus
HHV6, 7	human herpesvirus 6, 7
HUVEC	human umbilical vein endothelial cells
ICAM-1	intercellular adhesion molecule-1
IFNγ	interferon γ
IL-1α, -1β, -4, -5, -6, -7, -8, -12B, -15	interleukin-1α, -1β, -6, -7, -8, -12B, 15
ITCV	International Committee on Taxonomy of Viruses
IVIG	intravenous immunoglobulin
MCP-1, -2	monocyte chemoattractant protein-1, -2
MDMs	monocyte-derived macrophages
MIP-1α, -3	macrophage inflammatory protein-1α, -3
MHC	major histocompatibility complex
miRNA	micro RNA
MIG	monokine induced by gamma interferon
PAEC	porcine aortic endothelial cells
PAI-1	plasminogen activator inhibitor 1
PCMV/PRV	porcine cytomegalovirus/porcine roseolovirus
PTEC	proximal tubular epithelial cells
SuBHV2	suid betaherpesvirus 2
TCR	T cell receptor
TGF-β1	transforming growth factor β1
TF	porcine tissue factor
TNFα	tumor necrosis factor α
tPA	tissue plasminogen activator
